# Substance Flow Analysis: A Case Study of Fluoride Exposure through Food and Beverages in Young Children Living in Ethiopia

**DOI:** 10.1289/ehp.1002365

**Published:** 2010-12-06

**Authors:** Marian Kjellevold Malde, Ruth Scheidegger, Kåre Julshamn, Hans-Peter Bader

**Affiliations:** 1 National Institute of Nutrition and Seafood Research, Bergen, Norway; 2 Eawag, Swiss Federal Institute of Aquatic Science, Dübendorf, Switzerland

**Keywords:** defluoridation, endemic fluorosis, Ethiopia, Rift Valley, substance flow analysis, Wonji Shoa Sugar Estate

## Abstract

**Context:**

Dental and skeletal fluorosis is endemic in the Ethiopian Rift Valley. Children are especially vulnerable to excessive fluoride intake because their permanent teeth are still being formed. Strategies to reduce the total fluoride intake by children are thus warranted.

**Case presentation:**

By combining the results of field studies in Ethiopia, the relevant pathways for fluoride intake have been identified in 28 children 2–5 years of age living in two villages on the Wonji Shoa Sugar Estate in the Ethiopian Rift Valley. The focus of the present study was to simulate the fluoride intake of the children using the methods of material flow analysis (MFA) and substance flow analysis.

**Discussion:**

With a model based on MFA, we quantified the potential reduction in total fluoride intake given different scenarios—for example, by reducing the fluoride intake from drinking water and cooking water. The results show clearly that only by removing fluoride completely from both drinking and cooking water does the probability of remaining below the daily tolerable upper intake level exceed 50%. Both prepared food and food ingredients must be taken into consideration when assessing the total fluoride intake by children living in high-fluoride areas.

**Relevance:**

This knowledge will help health personnel, the government, and the food authorities to give scientifically based advice on strategies for reducing the total fluoride intake by children living in high-fluoride areas in the Ethiopian Rift Valley.

Until simple and low-cost defluoridation methods for use at the household level are fully developed, the excessive dietary intake of fluoride will continue to be a health problem in low-income countries. Fluoride is the 13th most abundant element found in the earth’s crust, and at least traces of it are found in all food and beverages (Smith and Ekstrand 1996). The fluoride content of drinking water consequently varies from trace amounts to toxic concentrations. The highest concentrations are generally found in groundwater, ranging from 1.5 to 36 mg/L in the Ethiopian Rift Valley (Tekle-Haimanot et al. 1987). The World Health Organization (WHO 2008) has recommended a guideline value of 1.5 mg/L in natural fluoridated drinking water. However, where intakes are likely to exceed 6 mg/day, it is appropriate to consider a local guideline fluoride concentration lower than 1.5 mg/L (WHO 2008). Fluorosis (chronic fluoride poisoning) is most easily detected in the teeth, in the form of mottling of the tooth enamel, and the two first years of life are most important to fluorosis development in the permanent central incisors, which are of most concern aesthetically (Hong et al. 2006). Skeletal fluorosis can be defined as excessive deposition of fluoride in bone (Saraux et al. 1994). Severe forms of skeletal deformities may develop as early as adolescence, and Cao et al. (2005) suggest that dental fluorosis can be a sign of early-stage skeletal fluorosis that might lead to full-scale debilitating skeletal fluorosis in adulthood. Although fluorosis cannot be cured, it can be prevented by reducing the fluoride intake.

In the Ethiopian Rift Valley, 41% of the drinking-water sources have a fluoride concentration exceeding 1.5 mg/L (Tekle-Haimanot et al. 2006), and food ingredients and food prepared with local water may also be a major fluoride source (Malde et al. 1997, 2004). Studies from the Rift Valley area indicate variations in the fluoride content of the same species (staple food), as well as between different growing areas. The relatively high fluoride concentrations found in some cereals and legumes analyzed by Malde et al. (1997) may be of practical importance because those food items are often central components in staple African food. Food was confirmed to be a major source of fluoride in a survey that assessed the fluoride intake in Ethiopian children using the duplicate diet technique. The results of this survey showed that high amounts of fluoride were retained in food prepared with high-fluoride water (Malde et al. 2004). The children investigated had a daily fluoride intake of 3.5 mg and 12.0 mg in two villages with drinking water containing 2 and 14 mg/L, respectively. Taking into account that the children’s permanent teeth are still under formation, this high fluoride intake is worrying, and strategies to reduce the fluoride intake are required.

## Case Presentation

By combining the results of field studies in Ethiopia (Malde et al. 1997, 2003, 2004), we have identified the relevant pathways for fluoride intake in 28 children 2–5 years of age living in two villages on the Wonji Shoa Sugar Estate (WSSE) in the Ethiopian Rift Valley. The focus of the present study was to simulate the fluoride intake of these children using the methods of material flow analysis (MFA). An MFA-based model allows the reduction in total fluoride intake for different scenarios to be quantified, for example, by reducing the fluoride intake from one or several dietary sources.

### Study area

WSSE is run by a state-owned company, the Wonji Shoa Sugar Factory (WSSF). The estate contains two factory villages and 13 plantation villages named with letters from A to M. Villages A and K were chosen for the food survey because of the differences in fluoride concentrations in their drinking water. The total population in WSSE at the time of the food survey was approximately 30,000. The WSSF provides free residential facilities, including housing, water supply and electricity, schools, and free medical services to its employees and their families. All villages are provided with well water for domestic purposes. Because of elevated fluoride concentrations in the drilled wells, defluoridation plants were installed between 1962 and 1976. The plants are not actually operational. At the time of the food survey, the pipeline supplying water to village A was broken, and the families collected drinking water from either Awash River (mean ± SD fluoride, 1.8 ± 0.3 mg/L) or from a well in the factory town, Wonji (2.1 ± 0.1 mg/L). The families in village K collected water from a well with a fluoride concentration of 14.4 ± 0.4 mg/L (Malde et al. 2003). The main staple food in the study area is injera made from teff (grain from the grass *Eragrostis tef*/*abyssinica*). Injera is unleavened bread prepared by fermentation of teff, wheat, barley, maize, or sorghum, or from a mixture of these (Stewart and Getachew 1962). Teff seeds have a high content of calcium and iron (Ågren et al. 1975).

### Ethical considerations

Ethical approval for the field study (Malde et al. 2003, 2004) was obtained from health and public authorities (Oromia region, Ethiopia). Informed consent was obtained from all families participating in the study. The procedures followed were in accordance with the ethical standards of the responsible committee on human experimentation and with the Helsinki Declaration of 1975, as revised in 1983 (World Medical Association 2008).

### Material and substance flow analysis

MFA and substance flow analysis are methods designed to account systematically for the material, substance, and energy use of a certain system. Based on an economic input–output analysis (Leontief 1936), they were originally developed in the chemical engineering sector. In the mid-1980s, these methods were further developed by Baccini and Brunner (1991) to account for the material, substance, and energy flows in whole regions. They were later extended by Baccini and Bader (1996) to include modeling concepts providing a systematic description and simulation of substance or material flows through a defined system. In the past two decades, these methods have been applied to many problems in different fields and on different scales. The material and substances studied vary from global resources (e.g., stone, wood, copper, phosphorus) to chemicals produced for modern daily life (e.g., pesticides, biocides, flame retardants, artificial sweeteners) (Neset et al. 2008; Schaffner et al. 2009). The procedure consists of four steps: system analysis, model approach, calibration, and simulation including sensitivity analysis and scenarios.

### System analysis

**Figure 1:**
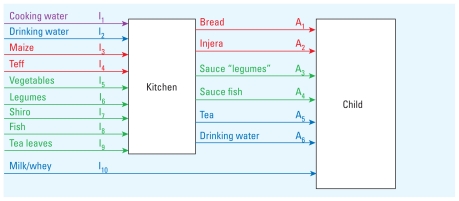
System analysis for fluoride intake in children living on the WSSE in the Ethiopian Rift Valley. The arrows denoted by *I*_1_ to *I*_10_ are the food and beverage ingredients used for cooking (input flows). These ingredients are “processed” in the kitchen to make prepared foods and beverages (*A*_1_ to *A*_6_) consumed by the children (consumption flows). Shiro is the Amharic name of pretoasted, crushed beans or peas mixed with spices. When it is prepared with berberre (local spices made of red peppers and various spices), it is called meten shiro.

The focus of this study was to systematically quantify the fluoride flows from water sources and food ingredients through meal and beverage preparation to the intake by the children living in the Ethiopian Rift Valley. During intensive field studies in Ethiopia (Malde et al. 1997, 2003, 2004), the relevant pathways for intake were identified. The following system analysis is the result of the system knowledge gained. We defined the system border as a household in village A or K with two “balance volumes” (the kitchen and the child), as shown in Figure 1. The flows can be divided into two groups: The consumption flows to the children and the “input” flows to the kitchen (Figure 1). The consumption flows cover the foods and beverages consumed. The input flows to the kitchen consist of all the “ingredients” needed to prepare the meals. In addition to the mass flows of the foods and beverages, fluoride and calcium flows and energy intake also were analyzed.

### Model approach

The goal of the substance flow model is to quantitatively trace fluoride intake back to the foods and beverages consumed, and further back to the ingredients used to prepare the foods and beverages. The key driving force of the system is the consumption pattern of the children—specifically, what types of foods, meals, and beverages are consumed, and in what amounts—which has been determined based on previous field studies (Malde et al. 1997, 2003, 2004). The ingredients (inputs) entering the kitchen are related to the foods and beverages by recipes [see Supplemental Material, Table 1 (doi:10.1289/ehp.1002365)]. Therefore, the model uses a “consumption-recipe” approach to describe the system most adequately.

### Consumption: recipe model

#### Consumption

The amount of consumed foods and beverages per day and child is known from field studies (Malde et al. 2003, 2004):


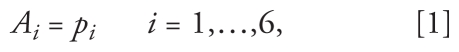


where *A*_1_, …, *A*_6_ are the food/drink flows and *p*_1_, …, *p*_6_ are the parameters representing the different foods/beverages consumed per day.

The input flow to kitchen according to recipe is as follows:


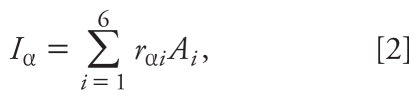


where *r*_α_*_i_* describes the fraction of an ingredient *I*_α_*_i_* in a food *A_i_*. This equation means that the ingredient (input) *I*_α_ is distributed in the kitchen among all foods *A_i_* according to the recipe for each food.

#### Related fluoride, calcium, and/or energy flows

The fluoride flow in an ingredient is related to the mass flow by concentration:


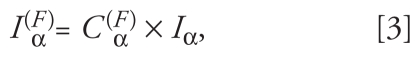


where *I*_α_^(^*^F^*^)^ is the fluoride flow in an ingredient *I*_α_ and *C*_α_^(^*^F^*^)^ is the concentration of fluoride in ingredient *I*_α_.

The fluoride in the food/drink flows is related to the consumed food/drink flows by the recipes and concentrations in the ingredients:


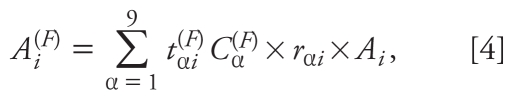


where *t*_α_*_i_*^(^*^F^*^)^ is the transfer function of fluoride contained in ingredient *I*_α_ to the food *A_i_* during cooking and *t*_α_*_i_*^(^*^F^*^)^ is a function of the fluoride concentration *C*_α_^(^*^F^*^)^ in ingredient *I*_α_, as well as the concentration of other elements in ingredient *I*_α_. Note that the expression *r*_α_*_i_* × *A_i_* is the amount of ingredient *I*_α_ in food/beverage *A_i_*. Summarized, the consumption recipe model is described by the following parameters: consumed food/beverage *p*_1_,..., *p*_6_, and recipes of the meals *r*_α_*_i_* the fluoride concentration *C*_α_^(^*^F^*^)^ of the fluoride in the ingredients and transfer function *t*_α_*_i_*^(^*^F^*^)^ through the kitchen. Clearly, the model is easily extendable to include other substances of concern. Each additional substance *X* is described by a set of parameters *C*_α_^(^*^X^*^)^ and *t*_α_*_i_*^(^*^X^*^)^ representing the concentration of *X* in each ingredient, and the transfer function for *X* from ingredient *I*_α_ to a food *A_i_*, respectively.

### Data used

Data describing the amount of food and beverages consumed, as well as the fluoride concentrations in the analyzed duplicate diets, have been published elsewhere (Malde et al. 1997, 2003, 2004). In the present analyses, we also included unpublished data on food intake and composition of the duplicate diets. When duplicate diets were being collected from the households, the mothers gave a detailed description of the ingredients in each food. The different food items (e.g., injera or bread) were weighed separately. On the basis of this information, therefore, we were able to specify the intake of the main foods in grams.

#### Data uncertainty

There are two main sources of uncertainty for the data collected: *a*) uncertainties caused by the measurement procedure (weighing of food, measuring of concentrations), and *b*) uncertainties regarding the recipes (i.e., uncertainties in description of compositions of a food). The estimated uncertainties for the different sources are as follows: According to the sampling procedure and chemical analysis, the relative standard deviation (RSD = SD/mean × 100%) of the fluoride concentrations is estimated to be 30% except for the concentration of fluoride in water. The RSD due to uncertainty in weighing is estimated to be about 30%. Recipes are based on information from a cookbook (Mesfin 1993), and we estimated the RSD to be 20%. The probability distributions of the data [see Supplemental Material, Table 1 (doi:10.1289/ehp.1002365)] are assumed to be truncated normal for the quantity of each food and beverage consumed (which may fluctuate but will always be > 0) and for the recipe-based proportions of ingredients in each food or beverage consumed (ranging from 0 to 100%), and lognormal for the measured concentrations of fluoride in each ingredient (consistent with chemical analysis data).

#### Food intake

The 50th and 90th percentiles of total food intake (wet weight) by the children (*n* = 28) were 452 ± 119 and 661 g/day, respectively (Malde et al. 2010). According to information sampled on the second day of the food survey, the children’s diets were composed of injera (made from teff, including fried injera, 42% of the total food intake), bread made of wheat, barley, maize, or a combination of these cereals (14%), sauce (typically containing legumes and/or vegetables, 22%), and “other” (primarily gruel or pasta, 22%). Because foods included in the “other” group were usually made from the same cereals used to make bread, the “bread” and “other” categories were merged in the MFA analysis. Based on these data, we are able to report approximate values for the intake of products made from teff (injera), products made from maize and wheat (e.g., bread, gruel), and sauce (mainly made from legumes and/or vegetables). The fluoride concentrations of the different food ingredients were based on data from published articles (Malde et al. 1997, 2006), and we took information on the moisture content of different prepared foods, and the energy and calcium content of both ingredients and prepared foods, from food composition tables for use in Ethiopia [Ethiopian Health and Nutrition Institute (EHNRI) 1997, 1998]. No data exist for the transfer functions *t*_α_*_i_*^(^*^F^*^)^. Therefore, as a first approximation, we assumed that all the fluoride is transferred to the dishes during the cooking process, that is, *t*_α_*_i_*^(^*^F^*^)^ = 1 (for further discussion of this assumption, see “Discussion”). Mean values used to estimate fluoride intake according to consumption of different foods and beverages (kilograms/child/day), proportions of different ingredients in each food/beverage consumed (percent), and fluoride concentrations in each ingredient and in drinking and cooking water (by village) are provided in Supplemental Material, Table 1 (doi:10.1289/ehp.1002365).

## Simulation

**Figure 2:**
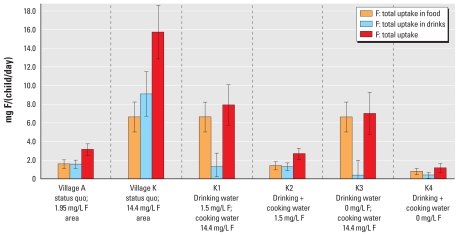
Daily estimated mean ± SD fluoride (F) intakes contributed by food and beverages, and total intakes: results according to observed fluoride concentrations in water sources for villages A and K and for four scenarios in village K (K1–K4).

**Figure 3:**
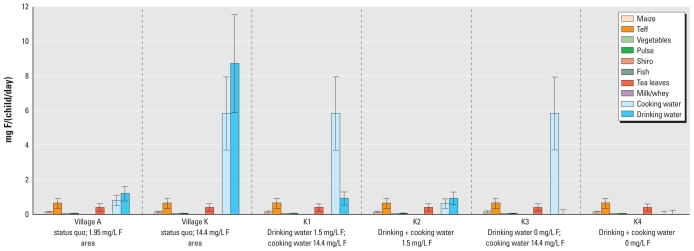
Daily estimated mean ± SD fluoride (F) intakes contributed by ingredients used in children’s food and drinks: results according to observed fluoride concentrations in water sources for villages A and K and for four scenarios in village K (K1–K4). Foods such as fish sauce were not consumed during the period observed but are part of the diet.

**Figure 4:**
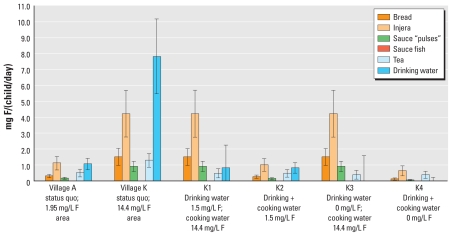
Daily estimated mean ± SD fluoride (F) intakes contributed by different food and drink items: results according to observed fluoride concentrations in water sources for villages A and K and for four scenarios in village K (K1–K4). Foods such as fish sauce were not consumed during the period observed but are part of the diet.

**Figure 5:**
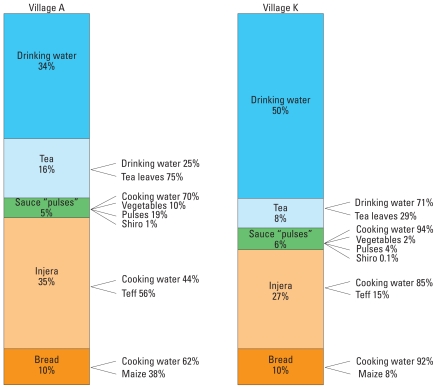
Relative contributions of prepared foods, beverages, and their ingredients to estimated daily fluoride intake in children from villages A and K.

We estimated fluoride intakes for children in villages A and K based on measured fluoride concentrations in water (1.95 mg/L and 14.4 mg/L in villages A and K, respectively) and under four alternative scenarios assuming reductions in the fluoride concentration of drinking water only, or reduced fluoride concentrations in both drinking water and cooking water (Table 1). In addition, we assumed total food consumption at either the 50th percentile or the 90th percentile for the study population and estimated calcium intake (50th percentile, 269 ± 69 mg/day; 90th percentile, 384 ± 100 mg/day) and energy intakes (50th percentile, 863 ± 253 kcal/day; 90th percentile, 1,258 ± 369 kcal/day) based on MFA, assumed to be the same in both villages. For village A, we estimated the total fluoride intake for each child to be 3.1 ± 0.6 mg/day (Figure 2). Cooking water, drinking water, and teff were the main fluoride sources into the “kitchen” (Figure 3), and prepared injera and drinking water contributed approximately the same amount to each child’s daily fluoride intake (Figures 4,5). For village K, we estimated the total fluoride intake to be 15.7 ± 2.9 mg (Figure 2), with injera contributing about half as much fluoride as drinking water (Figures 4,5). When we assumed total food intake corresponding to the 90th percentile for the population, estimated total fluoride intakes in villages A and K were 4.3 ± 0.9 and 21.4 ± 3.9 mg, respectively.

Simulations assuming fluoride levels of 1.5 mg/L in drinking water and cooking water suggested that the total fluoride intake of each child would be reduced to 2.7 mg/day in both villages (results for village K given in scenario K2; Figure 2) and that removing all fluoride from drinking water and cooking water would reduce the total fluoride intake to 1.6 mg/day for children in both villages (results for village K given in scenario K4; Figure 2).

### Uncertainty analysis

We conducted a Monte Carlo simulation with a sample size of 100,000, assuming that data were distributed as shown in Supplemental Material, Table 1 (doi:10.1289/ehp.1002365). For village A, the distributions for uptake from food and uptake from beverages were almost the same, varying in a range from 0.3 to 3.5 mg fluoride/(child/day); the range for the total uptake was from 1.3 to 5.3 mg fluoride/(child/day). In contrast, for village K the uptake from food varied from 2 to 13 mg fluoride/(child/day); for beverages, from 1.5 to 17 mg fluoride/(child/day); and for total uptake, from 5 to 25 mg fluoride/(child/day).

The daily tolerable upper intake level (UI) of fluoride for children up to 8 years of age is 0.1 mg/kg body weight [Standing Committee on the Scientific Evaluation of Dietary Reference Intakes (SCSEDRI) et al. 1997]. The measured mean weight of the children in the present study was 13.0 ± 2.4 kg in village A and 13.7 ± 2.7 kg in village K (Malde et al. 2003), resulting in a UI of about 1.3 mg fluoride/(child/day) for children in both villages. From the assumed probability distributions for villages A and K, we estimate that < 1% of children in village A and none of the children in village K would have daily fluoride intakes below the UI of 1.3 mg/day (Table 1) (SCSEDRI et al. 1997). Only by removing fluoride completely from both drinking and cooking water would the probability exceed 50% for a child living in both villages to have intakes below the tolerable uptake level (Table 1). For all other scenarios, the probability of being below the tolerable uptake level for children is < 10% for children in village A and < 1% for children in village K.

## Discussion

The total fluoride intakes by the children from villages A and K estimated by the MFA model were 3.1 ± 0.6 and 15.7 ± 2.9 mg/day, respectively (Figure 2). These values are consistent with fluoride concentrations measured in duplicate diet samples [3.5 and 12.0 mg/day for villages A and K, respectively (Malde et al. 2003, 2004)]. This indicates a satisfactory agreement between the model and the chemically analyzed duplicate portions. The fact that the MFA result for village K was 25% above the measured fluoride intake suggests that fluoride is not completely transferred to foods during cooking when fluoride concentrations in cooking water are high. This means that the transfer coefficients *t*_1_*_i_*^(^*^F^*^)^ and *t*_2_*_i_*^(^*^F^*^)^ should decrease with increasing fluoride concentrations. For village K, the MFA estimates of the relative contribution of prepared food to total fluoride intake (42%) were in accordance with the chemically analyzed duplicate diet portions (40%). For village A, the MFA estimated intake of fluoride from prepared food was less than the value based on the chemically analyzed duplicate diet portions (50% and 63%, respectively). However, both values are within 1 SD of each other.

Of the food ingredients entering the “kitchen,” teff and tea leaves contributed fluoride (Figure 3) regardless of the fluoride concentration in the water source. Injera and tea made the greatest contribution to fluoride intake among the different prepared food/beverage groups, and injera prepared with high-fluoride water contributed significantly to the total fluoride intake in village K (Figure 4). This indicates that prepared food must be considered when assessing the total fluoride intake of children in high-fluoride areas. The high fluoride intake is in accordance with the reported incidence of dental fluorosis in children 12–15 years of age from the two villages, which was 92% and 100% in villages A and K, respectively (Wondwossen et al. 2004). Thus, the total fluoride intake by these children is far too high, and strategies must be taken to reduce the fluoride intake in order to reduce the prevalence of dental fluorosis, and possibly also the more severe form, skeletal fluorosis. High-quality bone char produced in Kenya can reduce the fluoride content of drinking water with a natural fluoride concentration as high as 23 mg/L (Samuel et al. 2009) to a concentration below the international WHO guideline of 1.5 mg/L (WHO 2008). Preliminary results show that it is accepted by the population in the Ethiopian Rift Valley and can be implemented both at household and community-based levels (Samuel et al. 2009). Our model suggests that reducing the fluoride concentration of drinking water to 1.5 mg/L (scenario K1, Figure 2) would reduce the total fluoride intake to 2.9 mg/day and 7.9 mg/day for children in villages A and K, respectively. Reducing the fluoride concentration in drinking and cooking water to 1.5 mg/L (scenario K2) would reduce daily fluoride intake to 2.7 mg/day in children in both villages. However, only by removing fluoride completely from both drinking and cooking water can there be > 50% possibility for the children living in both villages to have daily intakes below the daily tolerable intake level of 1.3 mg/day (SCSEDRI et al. 1997).

An adequate intake of protein and calcium has been shown in animal studies to protect against fluorosis (Ekambaram and Paul 2001; Maguire et al. 2005; Pius and Viswanathan 2008). In a study on young children from India, a diet low in calcium was associated with skeletal fluorosis, a more serious outcome than dental fluorosis alone (Khandare et al. 2005). According to Malde et al. (2004), the children whose data we used in the present study had low dietary intakes of energy, protein, and calcium. Measured calcium intakes based on duplicate diets were 270 mg/day and 370 mg/day in villages A and K, respectively (Malde et al. 2004), which is low compared with the recommended intake for this age group (500–550 mg/day) (Food and Agriculture Organization of the United Nations/WHO 2001) but above what can be considered to be the biological requirement (Prentice and Bates 1994). According to the MFA analysis, the dietary intake of calcium was 270 mg/day. This is in accordance with the analysis of the duplicate diet from village A but is lower than that found in village K. Because the food ingredients used by residents of both villages came mainly from the same market, it is possible that dietary intakes differed because the water in the two villages had different calcium contents. Chemical analyses of different water sources in the Ethiopian Rift Valley show that shallow wells have a higher calcium concentration than does surface water (Reimann et al. 2003). This may explain the difference in calcium intake between the two groups of children, because most of the families in village A collected their drinking water from the nearby Awash River, whereas the water source in village K was a well (Malde et al. 2003).

In summary, our case study suggests that both drinking water and water used for food preparation should have a fluoride concentration below the WHO guidelines. Even with a water fluoride concentration close to 0 mg/L, the total fluoride intakes of children in the study area would be 1.2 mg (at the 50th percentile of energy intake) and 1.6 mg (90th percentile energy intake) because of fluoride in the food ingredients. In village K, a reduction of the fluoride concentration to 1.5 mg/L will most likely not reduce the incidence of dental fluorosis but may be sufficient to prevent the development of skeletal fluorosis.

## Figures and Tables

**Table 1:** Probability of children’s estimated daily fluoride intakes (from beverages and foods) being below the UI of 1.3 mg/day^a^ with different scenarios where drinking water and/or cooking water is untreated, reduced to the WHO guideline of 1.5 mg/L, or reduced to 0 mg/L.

Village/scenario	Drinking water (mg/L)	Cooking water (mg/L)	Beverage (%)	Food (%)	Total (%)
Village A	1.95	1.95	30	29	0.02
Village K	14.4	14.4	0.01	0.002	0
Scenario A1	1.5	1.95	55	28	0.1
Scenario K1	1.5	14.4	53	0.002	0.1
Scenario A2 and K2	1.5	1.5	55	45	0.2
Scenario A3	0	1.95	98	29	8.2
Scenario K3	0	14.4	72	0.002	0.6
Scenario A4 and K4	0	0	99	90	54

a The UI of fluoride for children up to 8 years of age is 0.1 mg/(kg body weight/day) (SCSEDRI et al. 1997). The measured mean weight of the children was 13 ± 2.4 kg in village A and 13.7 ± 2.7 kg in village K (Malde et al. 2003), giving a daily UI of about 1.3 mg for children in both villages.
